# Metabolic Profiling of Dividing Cells in Live Rodent Brain by Proton Magnetic Resonance Spectroscopy (^1^HMRS) and LCModel Analysis

**DOI:** 10.1371/journal.pone.0094755

**Published:** 2014-05-12

**Authors:** June-Hee Park, Hedok Lee, Rany Makaryus, Mei Yu, S. David Smith, Kasim Sayed, Tian Feng, Eric Holland, Annemie Van der Linden, Tom G. Bolwig, Grigori Enikolopov, Helene Benveniste

**Affiliations:** 1 Cold Spring Harbor Laboratory, Cold Spring Harbor, New York, United States of America; 2 Department of Anesthesiology, Stony Brook Medicine, Stony Brook, New York, United States of America; 3 Department of Applied Mathematics and Statistics, Stony Brook University, New York, United States of America; 4 Division of Human Biology, Fred Hutchinson Cancer Research Center, Seattle, Washington, United States of America; 5 Department of Biomedical Sciences, Bio-Imaging Laboratory, University of Antwerp, Belgium; 6 Neuropsychiatry Laboratory, Copenhagen University Hospital, Copenhagen, Denmark; 7 Department of Radiology, Stony Brook Medicine, Stony Brook, New York, United States of America; The Norwegian University of Science and Technology (NTNU), Norway

## Abstract

**Rationale:**

Dividing cells can be detected in the live brain by positron emission tomography or optical imaging. Here we apply proton magnetic resonance spectroscopy (^1^HMRS) and a widely used spectral fitting algorithm to characterize the effect of increased neurogenesis after electroconvulsive shock in the live rodent brain via spectral signatures representing mobile lipids resonating at ∼1.30 ppm. In addition, we also apply the same ^1^HMRS methodology to metabolically profile glioblastomas with actively dividing cells growing in RCAS-PDGF mice.

**Methods:**

^1^HMRS metabolic profiles were acquired on a 9.4T MRI instrument in combination with LCModel spectral analysis of: 1) rat brains before and after ECS or sham treatments and 2) RCAS-PDGF mice with glioblastomas and wild-type controls. Quantified ^1^HMRS data were compared to post-mortem histology.

**Results:**

Dividing cells in the rat hippocampus increased ∼3-fold after ECS compared to sham treatment. Quantification of hippocampal metabolites revealed significant decreases in N-acetyl-aspartate but no evidence of an elevated signal at ∼1.3 ppm (Lip13a+Lip13b) in the ECS compared to the sham group. In RCAS-PDGF mice a high density (22%) of dividing cells characterized glioblastomas. Nile Red staining revealed a small fraction (3%) of dying cells with intracellular lipid droplets in the tumors of RCAS-PDGF mice. Concentrations of NAA were lower, whereas lactate and Lip13a+Lip13b were found to be significantly higher in glioblastomas of RCAS-PDGF mice, when compared to normal brain tissue in the control mice.

**Conclusions:**

Metabolic profiling using ^1^HMRS in combination with LCModel analysis did not reveal correlation between Lip13a+Lip13b spectral signatures and an increase in neurogenesis in adult rat hippocampus after ECS. However, increases in Lip13a+Lip13b were evident in glioblastomas suggesting that a higher density of actively dividing cells and/or the presence of lipid droplets is necessary for LCModel to reveal mobile lipids.

## Introduction

Production of new neurons in the adult brain is associated with cognitive function, response to therapies, neurodegenerative diseases, and aging [1]–[3]. Life-long generation of new neurons in the hippocampus has now been firmly established for humans [4]; therefore, it is expected that the ability to track actively dividing cells in the live brain will impact diagnosis and therapy of a wide range of disorders of the nervous system. This becomes possible due to imaging technologies such as positron emission tomography (PET) in combination with radioactive ligands such as 3′-deoxy-3′- [18F]fluoro-L-thymidine (^18^F-FLT) [5], [6]. Newly synthesized DNA of proliferating cells incorporates ^18^F-FLT much the same way it incorporates halogenated thymidine analogs 5-bromo, or 5-chloro-2′-deoxyuridine (BrdU and CldU), thus enabling the use of PET for the detection of dividing cells, including neuronal progenitors [5], [7], [8]. Another imaging technique which has been tested for tracking of neurogenesis in the live brain is proton magnetic resonance spectroscopy (^1^HMRS) [9]–[11]. For example, a previous ^1^HMRS study applied singular value decomposition processing algorithms [10] and reported detection of neurogenesis in the hippocampus of the live rodent brain after repetitive electroconvulsive shock (ECS) treatments via increases in a lipid-like spectral signature resonating at 1.28 ppm [10], [12]. The origin of the 1.28 ppm signal in NPCs is thought to stem from mobile fatty acyl chains of triacylglycerides (TG), free fatty acids, and cholesteryl esters [13] residing in the cytoplasm or near the cell membranes of neural progenitor cells (NPCs). This possibility is supported by the association between intracellular lipid bodies either adjacent to the plasma membrane or within the cytoplasm and the ^1^HMRS TG signal in various tissues including brain [13]. It is also supported by the recent finding of high levels of lipogenesis correlating and being necessary for proliferation of NPCs in the hippocampus, perhaps reflecting the requirements for additional lipids to build the structural membranes of the progeny cells [14].

The study demonstrating an increase in the 1.28 ppm signal via ^1^HMRS in the live rodent hippocampus following ECS [10] led to discussions in regards to 1) specificity of the 1.28 ppm signal itself; for instance, it may be reflecting apoptosis which, while being an integral part of the neurogenesis cascade, would not necessarily correlate with division of neural progenitors [11]; 2) the non-parametric nature of the spectral processing algorithm used and lack of validation with other more widely used spectral processing methods [15]; 3) elimination of phase information of the fitted peaks by the SVD algorithm [16]; and 4) issues pertaining to assumptions inherent to the singular value decomposition algorithm itself [16]–[18]. To further investigate the link between the metabolic profiles of hippocampal cells, neurogenesis, and cell proliferation, we sought an alternative approach to evaluate the changes in metabolites in the affected brain.

Over the past decades, a number of spectral metabolite algorithms have been developed in an effort to estimate metabolite concentrations accurately and reliably under varying degrees of spectral quality. These algorithms are based on parameterization in frequency and time domains [22]–[24], wavelet transform [25], and approaches such as principle component analysis [26]. In particular, we focused on a widely used frequency domain fitting algorithm, LCModel [22], because it is based on a fully automated software, calculates spectral signal-to-noise ratio (SNR) and spectral line-width, is relatively user non-interactive in regards to fitting parameters and has been widely employed in both animal and human ^1^HMRS studies [27]–[29]. In addition, the algorithm calculates Cramer-Rao lower bounds as an error estimate [22]. By using a set of phase modulated frequency peaks measured *in vitro* as prior knowledge, LCModel constrains fitting parameters consequently speeding up computation and enables faster estimation of metabolite concentrations [30], [31].

We now apply LCModel as an alternative spectral fitting algorithm to characterize the ^1^HMRS metabolic profiles of the live adult rodent brain before and after repetitive ECS and associate them with histological evidence of cell division. In addition, we apply the same methodological approaches to metabolically characterize glioblastomas (GBMs) [19] growing in genetically-engineered RCAS-TVA-J12p16/M9Pten mice. In these tumors dividing cells are abundant and lipid signatures may be amplified by the presence of intra- or extracellular lipid droplets as has been reported for other brain tumor models [20], [21]. Our results support the use of LCModel approach for analyzing the metabolic changes in the adult brain upon stimulation or abnormal cell division. Raw and processed data obtained in the present study are available (http://www.hedoklee.com) to all interested investigators for further analysis and processing using alternate spectral analysis approaches.

## Materials and Methods

The experiments were carried out in strict accordance with the recommendations in the guide for the Institutional Care and Use of Laboratory Animals (IACUC) of Brookhaven National Laboratory (IACUC #375), Stony Brook University (IACUC #227884-4), and Cold Spring Harbor Laboratory (IACUC #12-09-05-15), who approved the study. All surgical procedures including electroconvulsive shock were carried out under sodium pentobarbital anesthesia, and all efforts were made to minimize suffering. For the **ECS** experiments, adult female Sprague-Dawley (SD) rats (Taconic, Inc.) were used. The animals were randomly assigned to two treatment groups: Group 1 (n = 10) were exposed to ECS, and Group 2 (n = 10) were exposed to sham treatment. All animals received a baseline ^1^HMRS scan and one month later (age 12–14 weeks) were exposed to either ECS or sham treatments for five consecutive days. The day after the last treatment, the animals received an i.p. injection of CldU (128 mg/kg, i.p.) to label dividing stem and progenitor cells and then subjected to a repeat ^1^HMRS scan -. Twenty-four hours later, the animals received an overdose of pentobarbital (200 mg/kg) followed by perfusion fixation with paraformaldehyde and brains were analyzed for cell division, differentiation, and apoptosis (see details below). For the **GBM** mouse model, we used a genetically-engineered RCAS-TVA-J12p16/M9Pten mouse model [19] in order to compare the magnitude of lipid resonances at 1.3 ppm with histological evidence of apoptosis and cell division, as well as the presence of intra- and extracellular lipid droplets which are present in some brain tumors when necrosis predominate [20], [21]. The RCAS-TVA-J12p16/M9Pten mouse models are based on the RCAS/t-va technology, where transgenic mice carrying a viral receptor produced selectively in nestin-expressing cells are intracranially injected by a suspension of cultured mammalian cells expressing genes that can result in malignant cell transformation, such as PDGF or Kras. Cells that receive the oncogene are those that express nestin and are assumed to carry stem/progenitor properties. By carrying out these procedures with mice with mutations in genes such as p53, ARF, or pTEN, it is possible to generate tumors that resemble human gliomas. ^1^HMRS scans were performed on nine RCAS-TVA-J12p16/M9Pten mice and five wild-type mice to compare metabolic profiles of normal and GBM tissue. The mice were injected with BrdU (150 mg/kg, i.p.) two hours prior to euthanization with an overdose of pentobarbital and the brains processed for histology (see below).

### Electroconvulsive shock (ECS) and sham treatments

ECS was delivered daily for five consecutive days using a pulse generator (ECT Unit 57800, Ugo Basile, Comeriao, Italy). Sham-treated controls were handled identically to the ECS-treated rats, but no electrical shock was administrated. A short-term (30 min) anesthesia was attained by methohexital sodium (30 mg/kg, i.p.; Brevital sodium, JHP Pharmaceuticals), a barbiturate anesthetic. Immediately after the rapid anesthetic induction, a non-paralyzing dose of succinylcholine chloride (0.5–1 mg/kg, Sigma), a neuromuscular blocker, was injected into the femoral muscle 3 min before ECS, to reduce the otherwise high mortality due to ECS. Bilateral ECS was applied by clamping both ears via ear clips dampened in saline to enhance the electric conductance, with 100 Hz of pulse frequency, 0.5 msec of pulse width, 0.5 sec of shock duration, and 50 mA of current. Three rats died from respiratory arrest immediately after induction of anesthesia during the third day of treatment and were excluded from analysis.

### ^1^HMRS, anesthesia and monitoring

#### Rats

**Figure 1:**
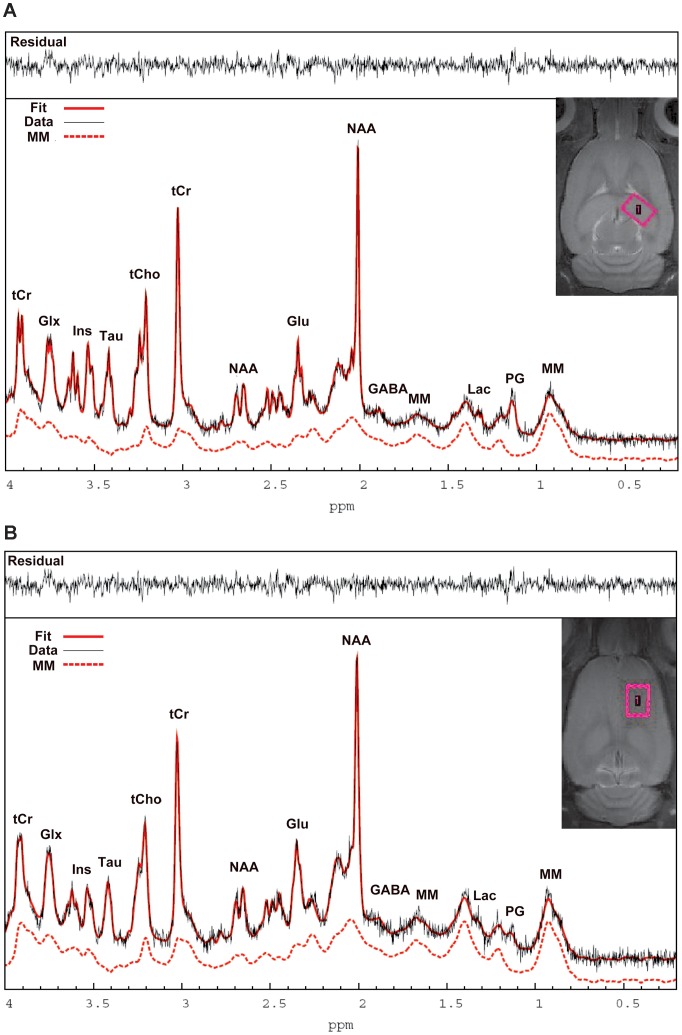
^1^HMRS spectra from the rat hippocampus and cortex analyzed with LCModel. Fig. 1A: The position of the hippocampal voxel is shown on T2-weighted MRI in the top right corner. The corresponding *in vivo* ^1^HMRS spectrum is shown analyzed by LC Model. Fig. 1B: The position of the cortical voxel is shown on the T2-weighted MRI as well as the *in vivo* corresponding ^1^HMRS spectrum. The two spectra shown are of high quality with spectral resolution necessary to resolve the labeled metabolites. Specifically, the raw unsmoothed spectra are shown (black),the LCModel fitted spectral output (red solid lines) and the corresponding macromolecular (MM) scan (dotted red lines) measured in the same animal using a PRESS metabolite nulled inversion-recovery pulse sequence in addition to the residual signals (top spectra). tCr = total creatine; Glx = glutamate + glutamine; Ins = myo-Inositol; Tau = Taurine; tCho = total choline; NAA = N-acetyl-aspartate; Glu = glutamate; MM = macromolecules; Lac = Lactate; PG =  Propylene glycol.

For imaging, the rats were anesthetized with pentobarbital (Nembutal, 40 mg/kg, i.p.) and allowed to breathe spontaneously during the ^1^HMRS experiment. Supplemental oxygen (2 L/min) was supplied via a snout mask. An antisialogue - glycopyrulate (0.01–0.02 mg/kg, i.p.) mixed with 0.9% NaCl (4 cc/kg) - was also administered to prevent excessive build-up of airway secretions and bradycardia during imaging. Physiological parameters including respiratory rate, oxygen saturation, body temperature, and heart rate were continuously monitored using MRI compatible optical monitors (SA Instruments, Inc., Stony Brook, NY). Body temperature was kept strictly within 36.5–37.5°C during imaging using an automated heating system (SA Instruments, Inc., Stony Brook, NY). All ^1^HMRS acquisitions were performed on a 9.4T/20 MRI instrument interfaced to a Bruker Advance console and controlled by Paravision 5.0 software (Bruker BioSpin, Billerica MA). For signal detection, a custom-made 3-cm surface radio-frequency coil placed under the rodent' head was used as a receiver and an 11.2-cm diameter volume coil (Bruker) was used as a transmitter. Localizer anatomical T2-weighted images were obtained in three orthogonal planes using a rapid acquisition with relaxation enhancement (RARE) sequence (TR = 2500, TE = 40 ms, NA = 2, RARE factor = 8, number of slices = 25, in plane resolution  =  0.117 mm/pixel, slice-thickness = 0.8 mm, slice gap = 0.1 mm) to assure correct positioning. First and second order shims were accomplished using MAPSHIM (Paravision 5.0, Bruker BioSpin, MA). For spectroscopy, two rectangular single voxel volumes (3.5×2.2×2.5 mm^3^) positioned in 1) the hippocampus that included the subgranular layer of the dentate gyrus (DG) area, and the 2) somatosensory cortex (**Fig. 1**) were excited using a point-resolved spectroscopy (PRESS) sequence. The following MR parameters were used: TR = 4000 ms, TE = 12 ms; NA = 512; spectral width = 8012 Hz, number of acquired complex points = 4096 yielding a spectral resolution of 1.96 Hz/pts. Outer volume suppression was carefully applied using a hyperbolic secant pulse (bandwidth = 10125 Hz) in order to avoid lipid contamination from the skull. Retro frequency lock was active during all acquisitions for monitoring of the unsuppressed water signal within the same voxel volume and to correct for possible center frequency drift over the 34 min scan acquisition time. Data were acquired in single-scan mode and each free induction decay (FID) was therefore saved separately. In this series, a water unsuppressed scan was also acquired to serve as a concentration reference. **Mice**: All mice were anesthetized with 1–2% isoflurane delivered in a 1∶1 Air:O_2_ mixture and were allowed to breathe spontaneously. Physiological monitoring was performed as described for the rats above. Localizer anatomical T2-weighted images were obtained in three orthogonal planes using a RARE sequence (TR = 2500, TE = 40 ms, NA = 2, RARE factor = 8, number of slices = 23, in plane resolution = 0.10 mm/pixel, slice-thickness = 0.5 mm, slice gap = 0.1 mm) to assess tumor volume and to position the voxel for ^1^HMRS in GBM-like tissue, and in corresponding anatomical locations in the wild-type control mice. For ^1^HMRS a PRESS sequence was used with the following MR parameters: TR = 4000 ms, TE = 12 ms, NA = 512, spectral width = 8012 Hz, number of acquired complex points = 2048 yielding a spectral resolution of 3.91 Hz/pts., voxel size  = 2×2×2 mm^3^. In the ^1^HMRS acquisitions from the GBM mice, the spectral voxel was positioned so that it predominantly contained tumor-like tissue in the mice with larger GBMs but invariably included non-tumor tissue in mice with smaller GBMs. Outer volume suppression was carefully applied using a hyperbolic secant pulse (bandwidth = 10125 Hz) in order to avoid lipid contamination from the skull.

### Spectral data processing

After frequency and phase correction of individual FID signal, a summed FID signal was constructed using in-house software written in MATLAB. A summed spectrum was then analyzed by LCModel [22] using a simulated metabolic profile based on TE = 12 ms PRESS sequence at 9.4T. No filtering was applied to the summed FID. The following metabolites were included in the simulated basis set: alanine (Ala), aspartate (Asp), creatine (Cr), phosphocreatine (PCr), γ-aminobutyric acid (GABA), glucose (Glc), glutamine (Gln), glutamate (Glu), glycerophosphorylcholine (GPC), phosphorylcholine (PCh), glutathione (GSH), myo-inositol (myo-Ins), scyllo-inositol (scyllo), lactate (Lac), N-acetylaspartate (NAA), N-acetylaspartylglutamate (NAAG), phosphoethanolamine (PE) and taurine (Tau). In addition, simulated spectra of mobile lipids (fatty acyl proton groups CH_2_, predominantly saturated methylene hydrogens; Lip13a, Lip13b and CH_3_ methyl hydrogen at Lip09) were included [32]. The LCModel analysis was performed on each of the TE 12 ms spectra from the chemical shift range of 0.0–4.0 ppm. The Cramer-Rao lower bounds (CRLB) were used to eliminate statistically unreliable values. Quantification of metabolites' concentration was performed by calibrating the metabolites signals relative to water unsuppressed signal, assuming parenchymal water concentration of 36 mM, also taking into account correcting for differences in receiver gains and number of acquisitions between suppressed and unsuppressed scans. A challenge unique to short echo time *invivo* ^1^HMRS lies in the ability to accurately parameterize a broad macro-molecular baseline [33] [28]. In the present study, we eliminated the need for parameterizing macro-molecular baseline by including a population averaged macro molecular baseline in the basis set. For this purpose, twelve metabolite-nulled spectra were acquired using the inversion-recovery PRESS sequence (TR = 4000 ms, TE = 12 ms, inversion time  = 950 ms).

The commercially available pentobarbital solution (Nembutal) produces specific spectral signatures in the brain during anesthesia due to its high content of propylene glycol (PG) which resonates at 1.13 ppm [34], [35]. Thus, in order to accurately identify metabolites from LCModel in the rats anesthetized with pentobarbital, the spectral resonances resulting from PG in particular also needed to be incorporated in the basis data set. Phantoms were therefore prepared containing 5 mM sodium pentobarbital balanced to pH = 7.2 dissolved in standard solvent with 4,4-dimethyl-4-silapentane-1-sulfonic acid (DSS) as reference for proper phase correction. The temperature of the phantom was maintained at 37°C and the acquisition parameters were the same as described for the *in vivo* experiments except for a TR = 10000 msec and a spectral bandwidth of 7882 Hz. The processed spectra from the Nembutal phantoms were included in the basis data set according to protocol [28]. Thus, the final LCModel basis data set used for analysis of all TE = 12 ms spectra obtained from rats thus contained brain metabolites, simulated lipids, PG, and macromolecules determined *in vivo*.

### Immunohistochemistry

Neural stem and progenitor cells and other dividing and apoptotic cells in the sections of the rodent brain using immunocytochemistry as described elsewhere [36], [37] were quantified from histologically prepared brain sections. After euthanasia with an overdose of pentobarbital (200 mg/kg, i.p.), the rodents were transcardially perfused with 200 ml of phosphate buffered saline (PBS) and 200 ml of 4% paraformaldehyde (PFA) in PBS. The extracted brains were additionally post-fixed in 4% PFA in PBS overnight at 4°C and stored in PBS with 0.1% sodium azide at 4°C until sectioning. The brains were dissected sagittal at 50 µm thickness by using a vibratome (Vibratome, St. Louis, MO) and a subset of brain sections from the right hemisphere, collected at a distance of 600 µm were subsequently used for immunohistochemisty. After rinses with PBS, the sections were denatured in 2 N HCl at 37°C for 1 hr, for detection of CldU incorporated proliferating stem and progenitor cells or for BrdU. The denatured sections were neutralized with 0.1 M borate, pH 8.0, twice for 20 minutes. The sections were rinsed with washing solution (PBS with 0.2% Triton X-100) and incubated for blocking and permeabilization in PBS with 2% Triton X-100 and 5% goat serum at room temperature (RT) for 2 hr. After rinses with washing solution, the sections were incubated, at 4°C overnight, in antibody solution (PBS with 0.2% Triton X-100 and 3% GS) containing primary antibodies; rat anti-CldU (Accurate Chemicals, OBT-0030; 1∶1,000 dilution), rabbit anti-GFAP (Dako, Z-0034; 1∶500 dilution), mouse anti-NeuN (Millipore, MAB-377; 1∶800 dilution), guinea pig anti-BLBP (Frontier Institute, BLBP-BP-Af291-1; 1∶400 dilution), rabbit anti-cleaved caspase-3 (Cell Signaling, #9661S, D175; 1∶400 dilution). Two hours after the primary antibody reaction extension at RT, the sections were rinsed with washing solution. The sections were incubated in antibody solution, at RT for 2 hours, with goat secondary antibody and fluorescent dye conjugates, Alexa fluor (AF, Invitrogen; 1∶400 dilution)-405, AF-488, AF-568, and AF-633, which recognize the corresponding primary antibodies. After rinses with washing solution, the sections mounted on gelatin-coated slide glasses were cover-slipped over fluorescence mounting media (DakoCytomation Fluorescent Mounting Medium, Carpinteria, CA) for confocal microscopy.

### Nile Red staining for lipid droplets and quantification

Brain sections (50 µm thick) were prepared from a) rats exposed to ECS (n = 7) or sham treatments (n = 8) and b) GBM mice (n = 3) and wild-type control mice (n = 2). Sections were stained with Hoechst33342 and/or propidium iodide (2 µg/ml) to visualize and assess cell nuclei and with the Nile red lipid stain to reveal lipid droplets [38]–[40]. Stock Nile red solution (Life Technologies, N-1142; 1 mg/ml DMSO) was filtered through a 0.22 µm syringe filter. The sections were incubated at RT for 1 hr in a diluted Nile red solution (0.1 µg/ml 150 mM NaCl) and then pretreated with RNase A (25 gµ/ml) to avoid staining cytoplasmic RNA during the blocking and permeabilization steps. After washing with PBS and mounting, the sections were examined by fluorescence microscopy. Numbers of normal appearing Nile red^+^ cells, dying or apoptotic Nile red^+^ cells, as well as extracellular Nile red^+^ lipid droplets in select sections within the ^1^HMRS voxel volume were quantified.

### Image capture

Following immunostaining, the brain sections were imaged using a spinning disk microscope and labeled NPC cells were counted using optical dissector stereologic technique [41]. All fluorescence immunostaining images were collected employing a Perkin-Elmer UltraVIEW VoX high speed spinning disk (Yokogawa CSU-X1) laser confocal microscope and Volocity software. All representative images were taken by Zeiss LSM710 and LSM780 and imported into Adobe Photoshop Version 12.0 (Adobe Systems Incorporated, San Jose, CA). Brightness, contrast, and background were adjusted using the “brightness and contrast” controls.

### Quantification of NPCs in adult rat brain

Quantitative analysis of cell populations was performed by means of design-based (assumption free, unbiased) stereology [41], [42]. Slices were collected using systematic-random sampling. One brain hemisphere was randomly selected per animal. The hemisphere was sliced in the sagittal plane in a lateral-to-medial direction, from the beginning of the lateral ventricle to the center, thus including the entire DG. The 50 µm slices were collected in 12 parallel sets, each set consisting of 10 slices, each slice 600 µm apart from the next. For cell quantification in the DG, cells of each type were counted in every slice, using the 40× objective. Adjacent z-stacks of the entire thickness of the section were collected by means of confocal microscopy. All of the cells in the section were counted, excluding those in the uppermost focal plane, and assigned to their corresponding category (CldU^+^; CldU^+^ GFAP^+^ radial glia-like stem cells; CldU^+^ GFAP^−^ amplifying NPCs). The average number of cells of each category from each slice was multiplied by 7.2 (average number of slices counted per animal from one set), and then multiplied by 24 (the number of sets of slices per animal from two hemispheres) to the value representing the total number of cells per rat; these numbers are reported.

### Quantification of the cell density and populations in GBM mice

We applied confocal microscopy to quantify cell density and evaluate cell phenotypes in GBM mice (n = 3) and wild-type control mice (n = 2). We used a spinning disk microscope (Perkin-Elmer) with a 60 x objective lens to focus on the areas corresponding to the ^1^HMRS voxel position. Stitched confocal images were obtained for 2 mm (x-axis) ×1 mm (y-axis) ×10 µm (z-axis) areas per section, and cells were analyzed within the voxel of ^1^HMRS using at least 2–3 brain sections per mouse. Stitched images were further cropped to a smaller size of 100 µm×100 µm×5 µm for the virtual counting via systemic and random selection. Cell density and the presence of lipid droplets were determined using staining with Hoechst33342 and Nile red respectively. Nile red-positive cells were characterized by intracellular lipid droplets with a diameter of less than 2 µm in the cytoplasm, whereas extracellular Nile red-positive lipid droplets were larger than 2 µm in diameter. Fluorescent signals of lipophilic Nile red dissolved in DMSO were obtained with an excitation wavelength of 530 nm and an emission wavelength of 575 nm. The density of BrdU-stained cells and pyknotic cells was determined by quantifying PI^+^ cells. Pyknotic cells were defined as cells, densely stained with PI, characterized by a compact shape and a condensed nucleus less than 3 µm in diameter. Cell number and density was determined in all the capture images in several views, including 3D opacity and Z plane using Volocity 3D image analysis software (Version 6.3).

### Statistical analysis

All data are presented as mean ± standard deviation. Statistical analyses were performed using SAS version 9.2 (SAS Institute Inc., NC, USA) and XLSTAT Version 2011 (Addinsoft, NY, USA) with a p-value <0.05 as statistically significant. Normality was examined using the Shapiro-Wilk test. Repeated measures analysis of variance (ANOVA) was performed to examine if the pre-selected metabolite concentration change from baseline to pre-op were different between the ECS or sham groups. Two-sided paired t-test was performed as post-hoc tests on all pre-selected quantified metabolites in order to examine how these metabolites changed between baseline scan and post-treatment scans within each group respectively, and between normal and tumor tissue in the mice studies.

## Results

**Figure 1** shows representative baseline ^1^HMRS spectra from the hippocampus (**Fig. 1A**) and the cortex (**Fig. 1B**) of a rat (no line broadening was applied) and the corresponding anatomical locations of the acquisition voxels in the rat brain. ^1^HMRS spectra from the hippocampus were of high quality as evaluated by the full width at half maximum (FWHM) and the signal-to-noise ratio (SNR) derived from LCModel which averaged 0.015±0.003 and 25±4, respectively. The cortical ^1^HMRS spectra were of inferior quality compared to those acquired in the hippocampus and five cortical spectra were discarded from the final analysis due to highly aberrant baseline LCModel fittings. FWHM and SNR for the remaining cortical spectra included in the analysis averaged 0.023±0.004 and 23±3, respectively. Based on an acceptable range of a given metabolite' average CRLBs, the concentration of the following metabolites ([metabolite]) could be quantified in the hippocampus and cortex: [Cr] (CRLB ∼11%), [PCr] (CRLB ∼7%), [GABA] (CRLB ∼9–16%), [Gln] (CRLB ∼7%), [Glu] (CRLB ∼3%), [GSH] (CRLB ∼15–30%), [Ins] (CRLB ∼7%), [NAA] (CRLB ∼2%), [PE] (CRLB ∼12%), [Tau] (CRLB ∼4%) and [GPC+PCh] (CRLB ∼10–15%). LCModel via simulation tracks the 1.28–1.30 ppm component previously found to be enriched in neural stem cells [10] and is referred to as ‘Lip13a+Lip13b’. However, analysis of baseline ^1^HMRS spectra from the hippocampus demonstrated that the CRLBs of Lip13a+Lip13b were undetectable (CRLB  = 999%) in 30% of the animals. For the cortex, 60% of the baseline CRLBs for Lip13a+lip13b were undetectable. Therefore, for the rat ECS and sham groups [Lip13a+Lip13b] are evaluated based on group averaged spectra of the animals before and after the ECS or sham treatments. Averaged group spectra for each condition was constructed by taking an average of the eddy current corrected and water scaled FID signals of each scan, which thereafter underwent LCModel analysis for metabolite quantification using the same basis data set as described previously.

### Effect of ECS on metabolites

**Figure 2:**
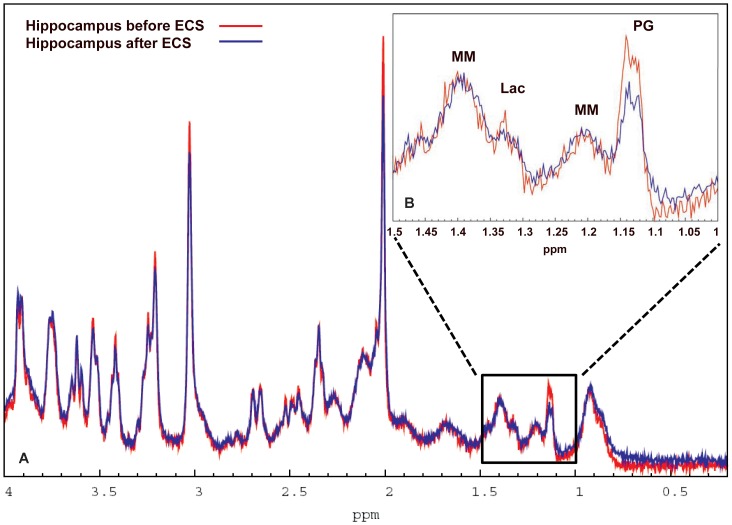
Summed and averaged ^1^HMRS hippocampal spectra before and after ECS. Fig. 2A: The summed and averaged baseline spectra acquired in the hippocampus from all the ECS rats (n = 7) are shown in red and the summed post-ECS spectra from the same rats are shown in blue. Note that the NAA peak is smaller in the summed post-ECS spectrum when compared to baseline summed and averaged spectra. Fig. 2B: Enlargement of the spectral profiles in the spectral range of 1.5 ppm–1.0 ppm from the summed baseline and post-ECS spectra are shown demonstrating that among the visible peaks, only propylene glycol appears to be different between the two conditions. NAA  =  N-acetyl-aspartate.

We first executed a repeated measures ANOVA on pre-selected metabolites (Glu, Ins, NAA, GPC+PCh and Cr+PCr) in the two groups to investigate if metabolite concentration changes from baseline to post-treatments scans were different via significance test for ‘group by time effect’. A two-tailed paired t-test was used to determine whether the mean metabolite concentrations were significantly different between the baseline and the post-treatment states. *Hippocampus*: In the ECS group, only the mean [NAA] difference was significantly different from zero. Specifically, the [NAA] significantly decreased from 5.7 mM to 5.4 mM after ECS exposure (p-value  = 0.001) (Table 1). In the sham group no differences were found. Given that the concentration estimations of [Lip13a+Lip13b] and [Lip09] were very low and in many animals undetectable (as evidenced by the CRLBs), baseline and post-ECS spectra were summed and averaged to increase overall SNR. In this analysis, [Lip13a+Lip13b] before and after ECS was 2.8 mM (CRLB 14%) and 2.0 mM (CRLB 27%), respectively. **Figure 2** shows the averaged pre- and post-ECS spectra from the hippocampus and also magnifies the spectral range from 1.5–1.0 ppm between the two conditions. It is clear from **Fig. 2** that the NAA peak is lower after ECS when compared to baseline. However, there is no apparent difference in the spectral 1.5-1.0 ppm between the two groups of animals suggesting subtle or no changes in mobile lipids (**Fig. 2**). In the sham group [Lip13a+Lip13b] was 2.2 mM (CRLB 14%) and 0 mM (CRLB 999%) before and after sham treatment, respectively. *Cortex*: Several cortical spectra were discarded due to poor spectral line width and/or SNR, and spectra were available for 5 rats from ECS group 1 and 7 rats from the sham group 2. Significant group by time effect was found for [NAA] (p-value = 0.0075). Similar to the hippocampus, [NAA] significantly decreased after ECS treatment compared to baseline, while the concentrations of remaining metabolites were unchanged (results not shown).

**Table 1:** Hippocampal metabolite concentrations before and after ECS or sham treatment.

Metabolite	ECS Group (n = 7)		Sham Group (n = 8)	
	BaselinemM	Post-ECSmM	Baseline mM	Post-Sham mM
**Glutamate**	6.37±0.52	6.32±0.50	5.85±0.50	5.90±0.83
**Myo-Inositol**	4.18±0.39	4.30±0.35	3.98±0.50	4.38±0.65
**N-Acetyl-Aspartate**	5.76±0.23	5.42±0.16*	5.58±0.41	5.66±0.68
**Glycerophosphorylcholine + Phosphorylcholine**	1.80±0.34	1.68±0.41	1.63±0.37	1.81±0.85
**Creatine + Phosphocreatine**	5.57±0.07	5.53±0.16	5.43±0.16	5.44±0.49

For the ECS as well as the sham group a two-sided paired t-test was performed to determine whether the mean metabolite concentration difference from baseline to post-treatment was significantly different from zero. Data are presented as mean ± standard deviation (SD). (*p<0.001).

### ECS increases cell proliferation in rat hippocampus

**Figure 3:**
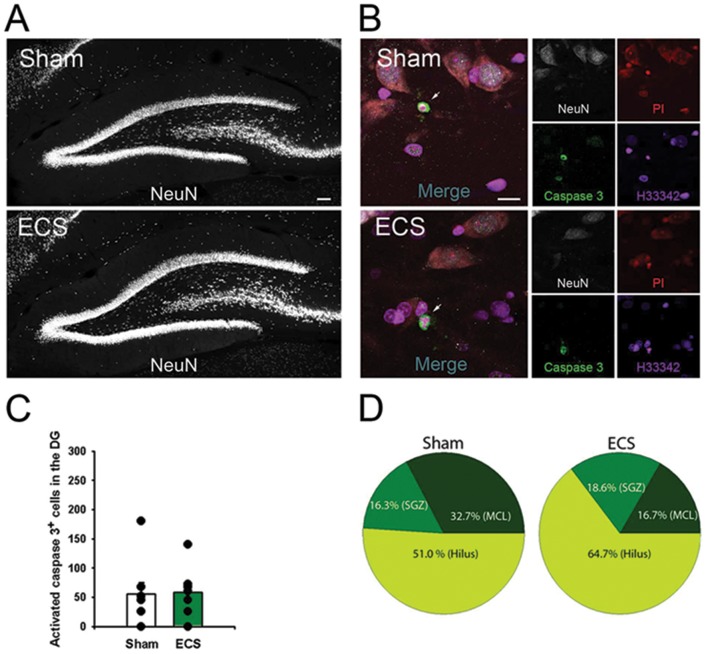
ECS does not induce neuronal loss. Fig. 3A: Representative images of the DG of sham and ECS-treated rats immunostained with neural marker NeuN. Fig. 3B: Representative images from sham and ECS-treated rats stained with anti-NeuN, anti-activated caspase 3, propidium iodide, and Hoechst33342. Arrows show activated caspase 3-positive apoptotic cells. Apoptotic cells stained for activated caspase 3-positive are characterized by compacted and shrunken nucleus as accessed by Hoechst33342 and PI. Fig. 3C: Histogram illustrating that there is no difference in the number of activated caspase 3-positive cells in the DG from sham (n = 8) and ECS (n = 7) rats. Fig. 3D: Distribution of activated caspase 3-labeled cells in the DG, illustrating that the hilus and inner molecular layer contains the majority of apoptotic cells. The SZG had very few apoptotic cells and the granular cell layer (GCL) did not show cells positive or activated caspase 3. Scale bars: Fig. 3A, 100 µm; Fig. 3B, 10 µm.

To examine the effects of ECS on cell proliferation in the adult hippocampus, we labeled newly synthesized DNA by injecting thymidine analog CldU. The total number of CldU^+^ cells in the DG increased significantly in rats exposed to repetitive ECS compared to the sham-operated rats (**Fig. 3B–D**) (ECS CldU^+^ cells  = 16771±2274 versus sham CldU^+^ cells  = 5823±577, p = 0.0003). The majority of CldU-labeled cells in both sham and ECS groups resided in the subgranular zone of the DG and corresponded to the transit amplifying rapidly dividing NPC (**Fig. 3B–C**). We also analyzed the brain sections for apoptotic cells, revealed by antibodies to activated caspase 3; there was no significant difference between the sham and the ECS groups (**Fig. 3C, D**). Finally we analyzed the brain sections for the presence of lipid droplets by Nile red staining; however, lipid droplets were absent both in the ECS- and sham-treated rats.

### RCAS-TVA-J12p16/M9Pten mice versus wild-type mice

**Figure 4:**
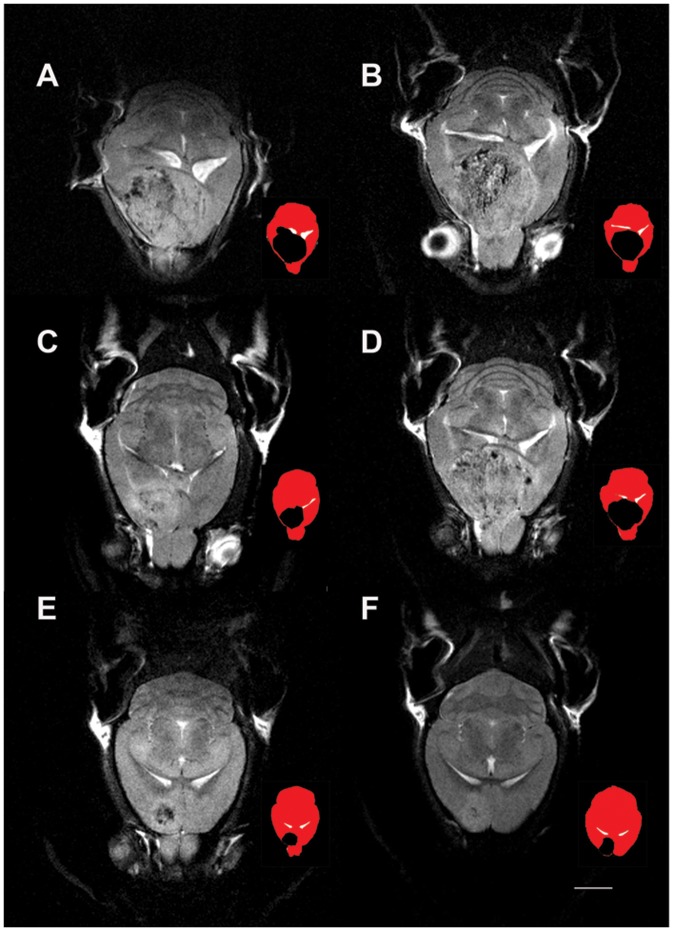
Glioblastoma size varies in the RCAS-TVA-J12p16/M9Pten mouse model. T2-weighted horizontal MRIs at the level of the lateral ventricles from four RCAS-TVA-J12p16/M9Pten mice with large glioblastomas (GBMs) (Fig. 4, A–D) and two RCAS-TVA-J12p16/M9Pten mice who developed smaller frontal GBMs) (Fig. 4, E–F) are shown. For each MRI image, a corresponding color-coded icon is shown to the right illustrating the GBM (black) in relation to ventricles (white) and the rest of the brain (red). As can be seen, the larger GBMs are compressing the lateral ventricles and incorporate both hemispheres. The T2-weighted MRIs of the mice with large GBMs demonstrate that the tumor tissue contain scattered areas associated with lower signal intensity and further that tissue at the edge of the tumor often is associated with brighter signal intensity. The smaller GBMs in Fig. 4E and F are located in frontal cortical areas and also contain tissue with mixed low and bright signal intensities. Scale bar  = 2 mm.

**Figure 5:**
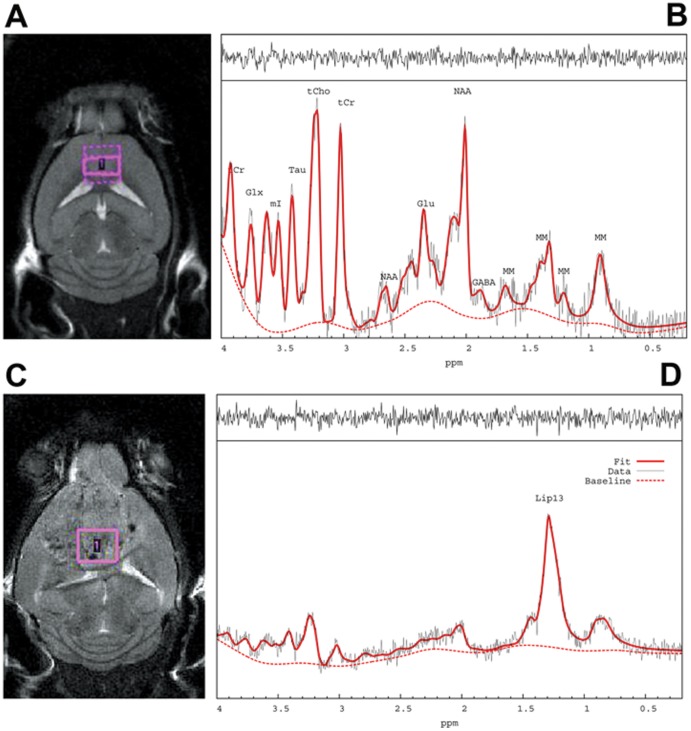
^1^HMRS spectra from glioblastoma are characterized by increased lipid signals. The ^1^HMRS voxel location on a T2-weighted MRI and the corresponding ^1^HMRS spectra from a control wild type mouse (Fig. 5 A, B) and a RCAS-TVA-J12p16/M9Pten mouse with large a glioblastoma (GBM) (Fig. 5 C, D) are shown. The ^1^HMRS spectrum from the GBM is dominated by the large and broad lipid peaks at 1.3 ppm and 0.9 ppm. The raw unsmoothed spectra are shown (black), the LCModel fitted spectral output (red solid lines). tCr = total creatine; Glx = glutamate + glutamine; Ins = myo-Inositol; Tau = Taurine; tCho = total choline; NAA = N-acetyl-aspartate; Glu = glutamate; MM = macromolecules; Lac = Lactate; GABA = gamma-aminobutyric acid.

**Figure 6:**
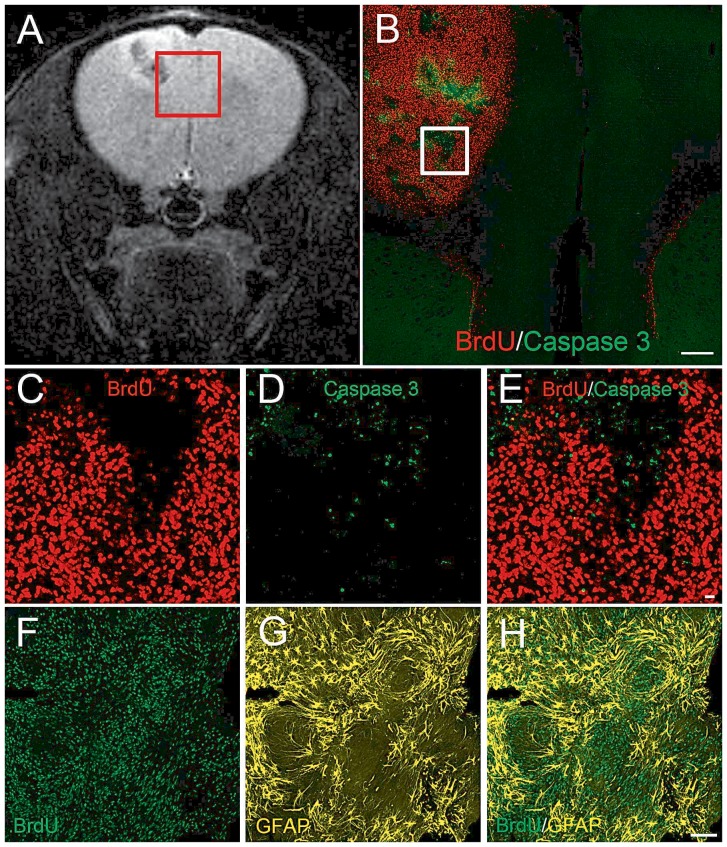
Immunohistochemistry of glioblastoma with elevated mobile lipid signal. To assess tumor growth, the RCAS-TVA-J12p16/M9Pten mice were injected with BrdU (150 mg/kg, i.p.) 2 hr before euthanasia. Fig. 6A: T2-weighted MRI of a RCAS-TVA-J12p16/M9Pten mouse who developed a small glioblastoma GBM in the left hemisphere. The region of the cerebral cortex (white inset) containing GBM and normal tissue was tracked and matched with histology (Fig. 6B–E). Fig. 6B: Immunostaining for BrdU and activated caspase 3, demonstrating that the GBM is filled with dividing cells mixed with islets of apoptotic cells. Fig. 6C–E: Higher magnification views of the inset in Fig. 6B illustrating the high density of dividing BrdU-positive cells intermingled with scattered apoptotic cells. (Fig. 6F–H) The GBM tissue immunostained for anti-BrdU and GFAP, a marker of neural stem cells and mature astrocytes. The images presented in Fig. 6B–H are single projection views based on 20-mm serial optical z-stacks. Scale bars: Fig. 6B, 1 mm; Fig. 6C–E, 10 µm; Fig. 6F–H, 100 µm.

**Figure 7:**
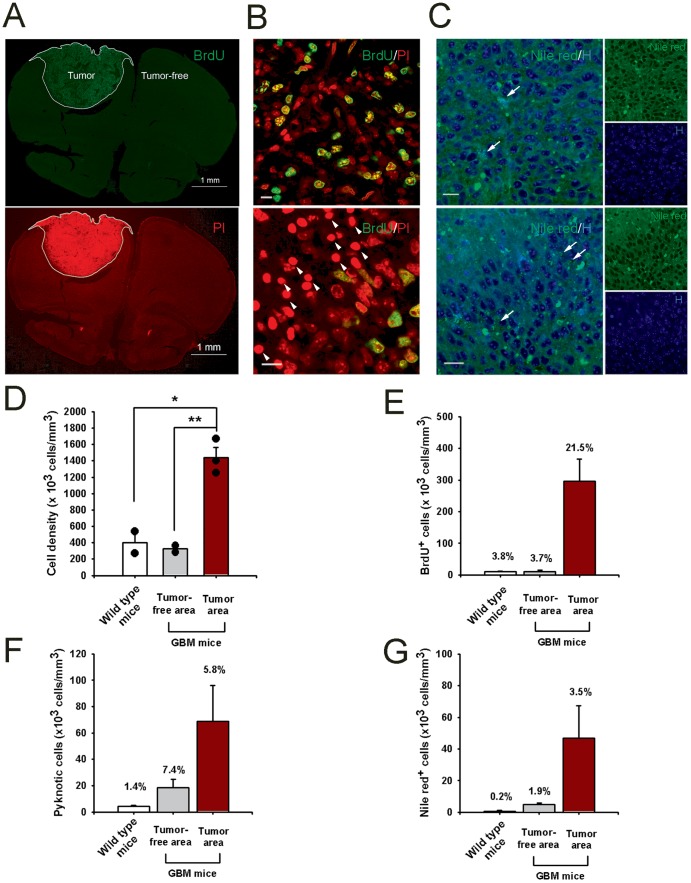
BrdU^+^ dividing cells and Nile red^+^ pyknotic cells in glioblastomas. Fig. 7A: Low magnification images of an RCAS-TVA-J12p16/M9Pten mouse with a glioblastoma (GBM), characterized by high cell density as demonstrated by counter-staining nuclei using PI. BrdU^+^ dividing cells are clearly evident within the tumor. Fig. 7B: Higher magnification images of the tumor, demonstrating high proliferative activity; BrdU^+^ dividing cells are distributed throughout the tumor (upper panel). Pyknotic cells are present in the very center of the tumor (lower panel). Pyknotic cells are characterized by PI staining as having smaller and more compact nuclei (lower panel). Scale bars, 10 µm. Fig. 7C: Higher magnification images of the tumor showing Nile red^+^ lipid droplets (visualized as small densely stained green structures). Nile red^+^ cells with intracellular lipid droplets (white arrows) and extracellular Nile red^+^ lipid droplets (red arrowheads) are clearly visible. The perimeter of the tumor (upper panel) is characterized by a smaller number of Nile red^+^ lipid droplets as compared to the center of the tumor (lower pane) suggesting that the latter contains more necrotic cells. Hoechst33342 (H), a nuclear counter-staining dye. Scale bars, 10 mµ; please note that in Fig. 7B–C representative images of cell density and populations are acquired at 0.5 µm of z stack depth. Fig. 7D: Cell density was determined by Hoechst33342 (H) staining. The tumor-enriched area is characterized by significantly higher cell density when compared to that of control mice or tumor-free tissue. Fig. 7E: Dividing cells were determined by BrdU immunohistochemisty and non-dividing cells by the nuclear counter-staining with PI. About 22% of cells were BrdU^+^ dividing cells in the tumor-enriched areas; and when correcting for cell density there were 40-fold more BrdU^+^ cells in the tumor when compared to non-tumor tissue. Fig. 7F: Pyknotic cells with small and compact nuclei are observed in the very center of the tumor. Approximately 5% of cells in the tumor-enriched area were pyknotic (20-fold more dead cells were observed in the tumor when compared to normal tissue). Fig. 7G: When accounting for cell density there were at least10-fold more lipid-containing cells in the tumor when comparing to non-tumor tissue.

We next examined the presence of the lipid [Lip13a+Lip13b] signals in the RCAS-TVA-J12p16/M9Pten mouse model of GBM [19], [43]. The T2-weighted MRIs revealed that the tumor size in the RCAS-TVA-J12p16/M9Pten mice varied greatly. Four mice had large tumors located in the forebrain which often extended from the olfactory bulb to the hippocampus and involved both hemispheres and the other 4 had smaller frontal positioned tumors (**Fig. 4**). The T2-weighted MRIs acquired from the mice with larger tumors revealed circular areas characterized by voxels with mixed low- and high signal intensities and the large GBMs compressed the lateral ventricles (**Fig. 4** **A–D**). ^1^HMRS spectra from the RCAS-TVA-J12p16/M9Pten mice were of varying quality due to magnetic field inhomogeneity, tumor size and ^1^HMRS voxel location. The average FWHM and SNR were 0.066±0.025 and 9±4, respectively. In control mice the ^1^HMRS spectra were of better quality as signified by an average FWHM and SNR of 0.036±0.007 and 9±1, respectively. **Figure 5** shows the ^1^HMRS voxel location on a T2-weighted MRI and the corresponding ^1^HMRS spectra from a control wild type mouse (**Fig. 5A, B**) and a RCAS-TVA-J12p16/M9Pten mouse with large GBM (**Fig. 5C, D**). The ^1^HMRS spectrum from the tumor is dominated by the large, broad lipid peak at 1.3 ppm and a smaller one at 0.9 ppm. The average CRLBs of [Lip13a+13b] was <20% which justified quantitative analysis. Quantitative analysis of [Lac], [NAA], [Lip13a+13b] and [Lip09] from normal and RCAS-TVA-J12p16/M9Pten mice with large tumors (N = 4) demonstrated that [Lac], [Lip13a+13b] and [Lip09] were significantly elevated while [NAA] was reduced in tumor tissue compared to normal brain (Table 2). **Figure 6** shows histology from one of the RCAS-TVA-J12p16/M9Pten tumors and demonstrates that in this particular tumor small islets of apoptotic cells are present; however, the majority of cells correspond to dividing BrdU^+^ cells. **Figure 7** shows examples of Nile red staining and the quantitative data from three of the RCAS-TVA-J12p16/M9Pten mice with tumors. The low magnification images from one of the mice with a smaller tumor demonstrates high density of dividing BrdU^+^ cells; furthermore, counter-staining of nuclei with PI clearly shows high cell density in the tumor compared to the normal surrounding tissue (**Fig. 7A**). The higher magnification images of the tumor-enriched area reveal increased cell proliferation activity, with a large number of BrdU^+^ cells distributed throughout the tumor (although some areas exhibited more dividing cells than others) (**Fig. 7B**, upper panel). In the very center of the tumor, pyknotic degenerated cells (characterized by their smaller and more compact nuclei) were evident (**Fig. 7B**, lower panel). The result of the Nile red staining is shown in **Fig. 7C**; where Nile red^+^ lipid droplets are seen as densely stained structures. Specifically, Nile red^+^ cells with intracellular lipid droplets and extracellular Nile red^+^ lipid droplets are observed within the tumor. The periphery of the tumor is characterized by a smaller number of Nile red^+^ lipid droplets when compared to the center of the tumor where necrotic cells are present (**Fig. 7C**). Quantification of cell populations on the brain sections from GBM (N = 3) and WT (N = 2) mice (within the voxel volume used for ^1^HMRS) revealed the following: a) cell density (as determined by Hoechst33342 staining) was ∼4-fold higher in the tumor compared to tumor-free tissue and/or control mice (**Fig. 7D**); b) within the tumor, ∼20% of the cells were BrdU^+^ and there were nearly 300-fold more dividing cells within the tumor when compared to non-tumor tissue (**Fig. 7E**); c) 4-fold more pyknotic cells were observed within the tumor when compared to non-tumor tissue (**Fig. 7F**); and d) there were 10-fold more cells with intra-cellular lipid droplets within the tumor when compared to cells in non-tumor tissue or wild-type control mice (**Fig. 7G**).

**Table 2:** Metabolite profile of WT and RCAS-TVA-J12p16/M9Pten with large glioblastomas.

Metabolite	WT (n = 5) mM	RCAS-TVA-J12p16/M9Pten (n = 4) mM
**Lactate**	3.39±1.23	8.02±3.16*
**N-Acetyl-Aspartate**	5.54±0.53	2.03±1.49**
**GPC+PCh**	1.96±0.24	1.45±0.98
**Lip13a + Lip13b**	1.23±1.44	24.75±21.75*
**Lip09**	0.63±0.64	5.44±3.27*

A two-sided independent t-test was performed to determine whether the mean metabolite concentration of WT and RCAS-TVA-J12p16/M9Pten mice with large tumors were significantly different. Data are presented as mean ± standard deviation (SD). GPC = Glycerophosphocholine; PCh = Phosphocholine. *p<0.05; **p<0.01.

## Discussion

In the present study, metabolic changes in the live rodent brain before and after repetitive ECS were assessed using ^1^HMRS acquired at 9.4T in combination with LCModel spectral fitting, and compared to quantitative immunohistological analysis of stem and progenitor cell proliferation in the DG of the hippocampus. We demonstrated that the total number of CldU^+^ cells (the majority of these representing amplifying NPCs) in the subgranular zone (SGZ) of the DG increased significantly (∼3-fold) in rats exposed to repetitive ECS compared to the sham group. The increase in hippocampal neurogenesis induced by ECS was associated with a 6% decrease in [NAA], however, we were not able to detect any other significant metabolic changes. Specifically, we did not document an increase in mobile lipids resonating at ∼1.28–1.30 ppm which has previously been shown to be enriched in neuronal stem cells *in vitro* and to increase in the live rodent brain after ECS when spectral processing was executed with singular value decomposition analysis to compute frequency spectra from time-domain MRS data [10]. We chose to do spectral processing with LCModel [22], because it has been extensively studied for its accuracy and reliability in both animal and clinical studies [27]–[29]. In addition, an experimentally acquired macro-molecular baseline which can influence the accuracy of the fit at ∼1.30 ppm was incorporated in the LCModel basis set. However, with these specific spectral processing conditions, we were not able to detect increases in the hippocampal Lip13a+Lip13b spectral signature in rats exposed to ECS when compared to baseline values. It is also noteworthy that we observed a decrease in [Lip13a+Lip13b] in sham treated rats when compared to baseline levels. Clearly, if baseline levels had not been obtained for each condition, a comparison of post-ECS and post-sham spectra would suggest that [Lip13a+Lip13b] increased after ECS. These results emphasize the importance of sham controls, but are difficult to interpret given the small sample size and the overall low [Lip13a+Lip13b] signal. It is possible that the overall quality (SNR and FWHM) of the hippocampal spectra acquired in the present study were inadequate for LCModel to reliably detect subtle changes in mobile lipids present in very low concentration ranges. Future studies focused on increasing spectral SNR using better hardware (*e.g*. cryo-probes, higher magnetic field strengths), more scan repetitions, and/or ultra-short TE pulse sequences will be able to address this issue. Alternatively AMARES [23], a prior knowledge-based algorithm using time domain signals, is also suitable for ^1^HMRS analysis and could be applied in future analysis. LCMODEL and AMARES have been reported to yield comparable performance as long as the underlying basis set is the same [44].

Ideally, the new ^1^HMRS data acquired before and after ECS analyzed with LCModel software should be compared with the SVD algorithm [10] to fully validate the sensitivity of LCModel in regards to detection of neurogenesis in the adult rat brain. In the absence of direct comparison of the LCModel derived data in this work with that of the previously used SVD algorithm, our study as such can neither support nor negate the previous SVD data obtained in rats exposed to ECS [10]. Our raw unprocessed spectra and LCModel processed data are available to all interested investigators for further analysis and processing using alternate spectral analysis approaches.

The quantitative ^1^HMRS analysis revealed that [NAA] was reduced by 6% in both the hippocampus (Table 1 and 2) and cortex after ECS when compared to baseline values. With the assumption that [NAA] reflects neuronal density, a decrease in [NAA] could indicate that the ECS procedure itself lead to some neuronal loss since there were no [NAA] changes observed in the sham controls. However, we did not observe an increased number of apoptotic cells in the hippocampus after ECS when compared to sham (**Fig. 3**). In human studies, investigators have reported that hippocampal [NAA] is unchanged after ECT [45], [46] and others have reported an increase in [NAA] in ECT responders [47]. In animal studies, metabolomic profiling with ^1^HMRS after ECS quantitative results have been reported in terms of metabolite ratios relative to total creatinine or NAA. For example, Sartorius *et al*., reported that the choline/NAA ratio increased after ECS treatment [48]. For comparison, in our study the choline/NAA ratio before and after ECS was unchanged. Biederman *et al*., have reported hippocampal changes in glutamate and choline in an animal model of treatment resistant depression (congenital learned helpless rats) after ECS, which also could not be confirmed in our study [49]. The differences in metabolic profiles reported previously and in the present study could be attributed to differences in animal strains, method of ECS and/or anesthesia regimen.

We also executed a separate series of ^1^HMRS experiments on mice with brain tumors infiltrated by actively dividing cells. Under these conditions metabolomic profiling with LCModel revealed an elevated, broad peak in the spectral range of 1.30 ppm as has previously been reported in other models of brain tumors by other investigators using LCModel analysis as well as other processing approaches [50]–[52]. High concentrations of lactate in brain tumors have also been reported and because the lactate –CH_3_ signal co-resonates with the methylene signals from mobile lipids at 1.3 ppm, it is often difficult to separate the two molecules using *in vivo* ^1^HMRS [20], [53]. In the RCAS-PDGF mice with large GBMs (n = 4) the lactate concentration averaged 8.3 mM which is in range with values reported in brain GBMs [54]. Additional spectral editing sequences such as two-dimensional MR spectroscopy [20], [53] are often used to make a conclusive assignment of lactate and lipid signals.

Metabolic profiling of human brain tumors using ^1^HMRS have revealed decreased levels of NAA, increased levels of choline, lactate, and mobile lipids [55]–[57]. Similarly, in preclinical studies, the amount of NAA was found to be lower in certain glioma models and the lipid 1.3 ppm peak significantly higher in particular in C6 glioma models [52]. The elevated lipid levels in brain tumors have been associated with various signatures including necrosis, membrane breakdown, cellular proliferation and/or apoptotic cells [13], [58]. Mobile lipids in tumors have also been associated with intracellular lipid bodies comprising mainly triglycerides [13].

In certain types of brain tumors including GBMs intra- and extra-cellular lipid droplets are present and have been associated with a higher than normal lipid signature by ^1^HMRS [20]. For example, in a recent study a C6 cell glioma rat model was used to characterize how metabolic signatures (via ^1^HMRS) of the tumor tracked over time compare with histological evidence of necrosis and lipid droplets [20]. In the early phases of tumor growth (7–14 days after C6 cell implantation) no lipid droplets and/or necrosis was present in the small-sized tumors and there was also no lipid signal detected by ^1^HMRS [20]. However, as the C6 GBM grows larger, necrosis and lipid droplets (0.2–2 µm) start to predominate and the area of necrosis is positively correlated with the corresponding magnitude of the lipid signature [20]. In the GBMs of RCAS-PDGF mice used in the present study, we also observed that a small fraction of dead or dying cells within the tumor contained lipid droplets (dying cells were present mostly in the very center of the tumor). Larger sized lipid droplets were also present in the extracellular space, again mostly in the very center of the tumor. Quantification of different cell types (i.e., BrdU^+^ proliferating cells, normal appearing non-dividing cells, and Nile red^+^ cells) within the area of the ^1^HMRS voxel used for the acquisition revealed cells (3%) that contained lipid droplets and a large amount of BrdU^+^ cells (20%). In other words, there are many more dividing cells than degenerating necrotic and apoptotic cells with lipid droplets in the tumor-abundant area and the mobile lipid signature may be derived from both of these entities.

We also observed decreased levels of NAA and increased levels of lactate and lipids. However, we could not confirm increased levels of choline as has been observed in other GBM animal models [51]. We also correlated the metabolic profiles of the GMBs with post-mortem evidence of cell proliferation (via BrdU) and apoptosis (via activated caspase-3 staining). The increased lipid peak was evident in both large and smaller GBMs and may be associated with the greatly increased cell division and/or a small fraction of lipid droplets. Noteworthy GBM cells carry numerous features of NPCs [59]–[61] and increased lipid signals may reflect high levels lipogenesis [14] in these rapidly dividing cells; however, it is also conceivable that oligodendrocytes in the tumor may contribute to the lipid signal.

In conclusion, our data show that increases in mobile lipids after ECS could not be confirmed in rats with evidence of a 3-fold increase in NPCs using *in vivo* ^1^HMRS and LCModel analysis. However, increases in the concentration of lipid signatures at 1.3 ppm and 0.9 ppm were evident in RCAS-PDGF mice with GBMs when compared to normal control mice.
