# Correction: Metabolic Profiling of Dividing Cells in Live Rodent Brain by Proton Magnetic Resonance Spectroscopy (^1^HMRS) and LCModel Analysis

**DOI:** 10.1371/journal.pone.0106127

**Published:** 2014-08-18

**Authors:** The PLOS ONE Staff

Figure 3 as it is currently published is incorrect. The published Figure 3 should be Figure S1 and will be replaced by a new figure 3 provided in this correction. This affects the following in-text citations within the Results and Discussion sections:

**Figure 3:**
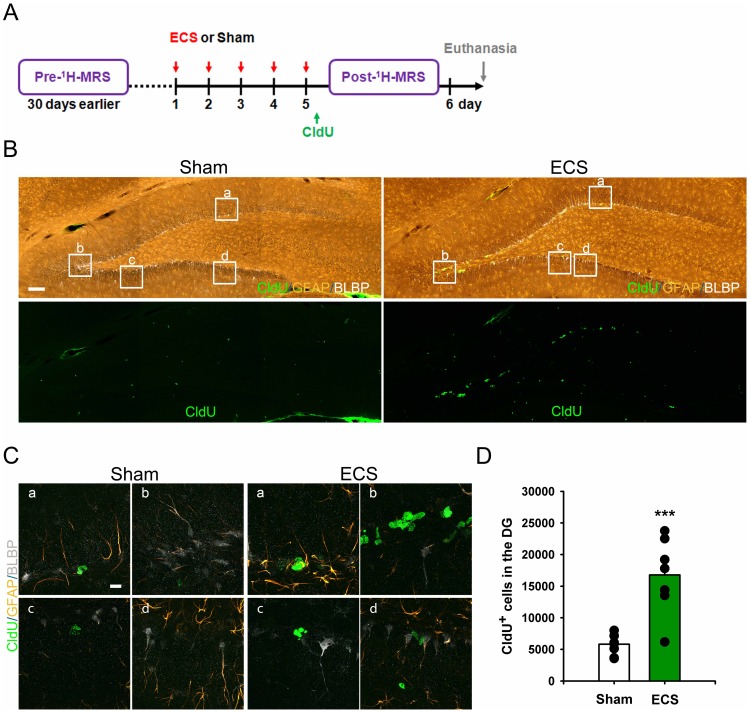
Cells labeled with CldU, GFAP and BLBP in sham- and ECS treated rats. **Fig. 3A**: Scheme of the experimental design specifying the injection of CldU in relation to 1HMRS scans, ECS or sham treatments and euthanasia. **Fig. 3B**: Representative confocal images encompassing the entire DG from rats exposed to sham or ECS treatment; and **Fig. 3C** shows higher magnification of insets selected from **Fig. 3B**. Note that ECS noticeably increases the number of CldU (green color) incorporated cells in the SGZ of the DG. The bodies of cells positive for glial-fibrillary-acid protein (GFAP, orange color) and brain lipid-binding protein (BLBP, grey color) are located in the superficial layer of the SGZ. The majority of CldU-positive dividing and newborn cells are present in clusters. **Fig. 3C**: Higher magnification images from a sham treated rat (a-d) and an ECS treated rat (a-d) each derived from the corresponding confocal images above. The higher magnification images again clearly shows more CldU+ cells in the ECS treated rats compared to sham controls. Scale bars: 100 µm in **Fig. 3B**, 10 µm in Fig. 3C. **Fig. 3D** shows the quantitative data for CldU+ cells in the DG demonstrating that ECS significantly increases the number of dividing cells. ***, p = 0.0003.

In the subsection “ECS increases cell proliferation in rat hippocampus” of the Results section, the fourth sentence should read, “We also analyzed the brain sections for apoptotic cells, revealed by antibodies to activated caspase 3; there was no significant difference between the sham and the ECS groups (Fig. S1 C, D).”

In the third paragraph of the Discussion section, the third sentence should read, “However, we did not observe an increased number of apoptotic cells in the hippocampus after ECS when compared to sham (Fig. S1).”

## Supporting Information

Figure S1 **ECS does not induce neuronal loss**. Fig. S1A: Representative images of the DG of sham and ECS-treated rats immunostained with neural marker NeuN. Fig. S1B: Representative images from sham and ECS-treated rats stained with anti-NeuN, anti-activated caspase 3, propidium iodide, and Hoechst33342. Arrows show activated caspase 3-positive apoptotic cells. Apoptotic cells stained for activated caspase 3-positive are characterized by compacted and shrunken nucleus as accessed by Hoechst33342 and PI. Fig. S1C: Histogram illustrating that there is no difference in the number of activated caspase 3-positive cells in the DG from sham (n = 8) and ECS (n = 7) rats. Fig. S1D: Distribution of activated caspase 3-labeled cells in the DG, illustrating that the hilus and inner molecular layer contains the majority of apoptotic cells. The SZG had very few apoptotic cells and the granular cell layer (GCL) did not show cells positive or activated caspase 3. Scale bars: Fig. S1A, 100 µm; Fig. S1B, 10 µm.
(TIF)
