# Supplementary figures and images for: Correction: Metabolic Profiling of Dividing Cells in Live Rodent Brain by Proton Magnetic Resonance Spectroscopy (1HMRS) and LCModel Analysis
Source: PLoS One. 2014 Aug 18;9(8):e106127. doi: 10.1371/journal.pone.0106127 (PMC4136929; doi:10.1371/journal.pone.0106127)

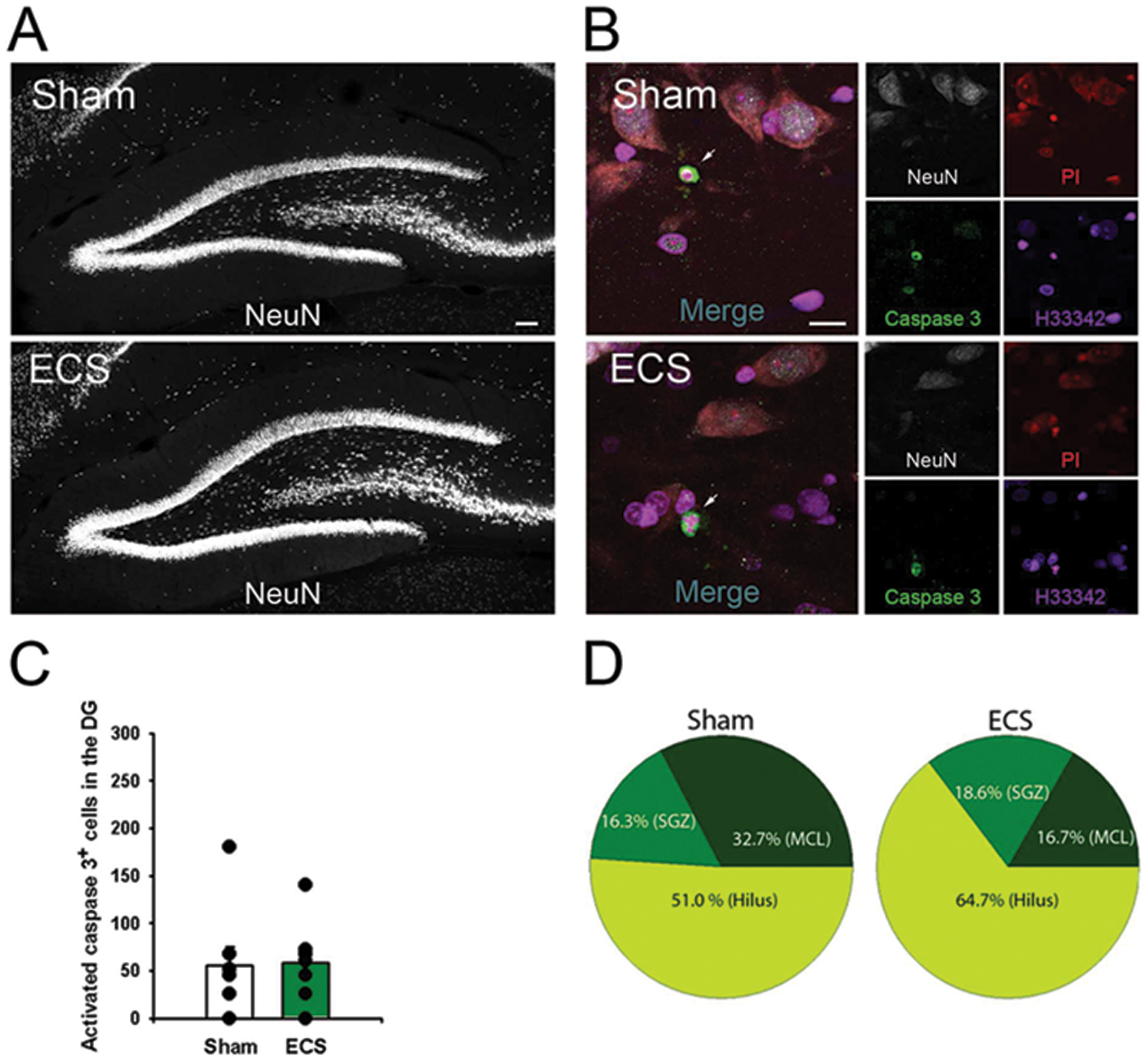

Supplement: Figure S1 — ECS does not induce neuronal loss. Fig. S1A: Representative images of the DG of sham and ECS-treated rats immunostained with neural marker NeuN. Fig. S1B: Representative images from sham and ECS-treated rats stained with anti-NeuN, anti-activated caspase 3, propidium iodide, and Hoechst33342. Arrows show activated caspase 3-positive apoptotic cells. Apoptotic cells stained for activated caspase 3-positive are characterized by compacted and shrunken nucleus as accessed by Hoechst33342 and PI. Fig. S1C: Histogram illustrating that there is no difference in the number of activated caspase 3-positive cells in the DG from sham (n = 8) and ECS (n = 7) rats. Fig. S1D: Distribution of activated caspase 3-labeled cells in the DG, illustrating that the hilus and inner molecular layer contains the majority of apoptotic cells. The SZG had very few apoptotic cells and the granular cell layer (GCL) did not show cells positive or activated caspase 3. Scale bars: Fig. S1A, 100 µm; Fig. S1B, 10 µm. (TIF) [file pone.0106127.s001.tif]
